# Valproic Acid Stimulates Hippocampal Neurogenesis *via* Activating the Wnt/β-Catenin Signaling Pathway in the APP/PS1/Nestin-GFP Triple Transgenic Mouse Model of Alzheimer’s Disease

**DOI:** 10.3389/fnagi.2019.00062

**Published:** 2019-03-26

**Authors:** Qinghua Zeng, Zhimin Long, Min Feng, Yueyang Zhao, Shifang Luo, Kejian Wang, Yingxiong Wang, Guang Yang, Guiqiong He

**Affiliations:** ^1^Chongqing Key Laboratory of Neurobiology, Chongqing Medical University, Chongqing, China; ^2^Department of Anatomy, Chongqing Medical University, Chongqing, China; ^3^Laboratory of Reproductive Biology, School of Public Health and Management, Chongqing Medical University, Chongqing, China; ^4^International Research Laboratory of Reproduction & Development, Chongqing Medical University, Chongqing, China; ^5^Department of Medical Genetics, Cummings School of Medicine, University of Calgary, Calgary, AB, Canada; ^6^Department of Biochemistry and Molecular Biology, Cummings School of Medicine, University of Calgary, Calgary, AB, Canada; ^7^Children’s Hospital Research Institute, University of Calgary, Calgary, AB, Canada

**Keywords:** Alzheimer’s disease, valproic acid, hippocampal neurogenesis, Wnt, GSK-3β

## Abstract

Alzheimer’s disease (AD) is an age-related neurodegenerative disease characterized by the deposition of amyloid-β (Aβ) peptides and neurofibrillary tangles (NFTs) and massive loss of neuronal cells in the brain. Adult hippocampus continuously generates new neurons throughout life to shape brain function and impaired neurogenesis may contribute to a series of cognitive deterioration associated with AD. Enhancing endogenous neurogenesis represents a promising strategy that may ameliorate AD-associated cognitive defects. However, neurogenesis-enhancing approaches and underlying mechanisms are still not well studied. Here, using a mouse model of AD amyloid precursor protein (APP/PS1/Nestin-GFP triple transgenic mice, 3xTgAD), we examined the effects of 4 weeks of valproic acid (VPA) treatment on hippocampal neurogenesis in 2- and 6-month-old mice. VPA treatment promoted cell proliferation and increased the density of immature neurons in the dentate gyrus (DG) of the hippocampus of 3xTgAD mice. Consistent with enhanced neurogenesis, behavioral and morphological analysis showed that VPA treatment improved the learning and memory ability of 3xTgAD mice. Mechanistically, VPA treatment increased β-catenin levels, accumulated the inactive form of glycogen synthase kinase-3β (GSK-3β), and induced the expression of NeuroD1, a Wnt target gene involved in neurogenesis, suggesting the activation of the Wnt signaling pathway in the hippocampus of 3xTgAD mice. This study indicates that VPA stimulates neurogenesis in the adult hippocampus of AD mice model through the Wnt pathway, highlighting VPA as a potential therapeutic for treating AD and related diseases.

## Introduction

Alzheimer’s disease (AD) is the most common form of age-associated dementia and shows increasing prevalence because of progressive aging of the population (Prince et al., 2016). It is characterized by multiple neuropathological features, such as the amyloid-β (Aβ) peptides aggregated into senile plaques (SPs), hyperphosphorylation of tau formed in the neurofibrillary tangles (NFTs), massive synaptic and neuronal loss that gradually leads to memory loss and cognitive impairment. The hippocampus, part of the limbic system important for learning and memory, is one of the earliest brain areas affected in AD (Mu and Gage, 2011; Rudnitskaya et al., 2019). The hippocampus is characterized by the continuous generation of new neurons throughout life (called adult hippocampal neurogenesis). Over the last several decades, a bidirectional relationship between memory and adult hippocampal neurogenesis has been evidenced (Deng et al., 2010; Friedland et al., 2001; Lazarov and Hollands, 2016). Accumulating evidence shows that alternations in hippocampal neurogenesis occur at the very early stage of AD pathology (Unger et al., 2016). The pool of new neurons being generated in the AD brain is decreased (Ekonomou et al., 2015), and an increase in neurogenesis might improve the spatial memory ability of experimental animals (Markiewicz et al., 2011; Sahay et al., 2011). Neural stem cells (NSCs) with the function of neurogenesis are found mainly in two places regions in the adult brain, that is the subventricular zone (SVZ) around the lateral ventricles (LVs) and the subgranular zone (SGZ) of the dentate gyrus (DG). Neurogenesis of NSCs and survival of newly differentiated cells make up for the lost neurons (Ming and Song, 2011; Fitzsimons et al., 2014; Gonçalves et al., 2016). Consequently, a promising treatment avenue of AD is to promote neurogenesis in the affected tissue by stimulating NSCs and neural progenitor cells (NPCs) in the neurogenic niches.

Valproic acid (VPA), a first-line antiepileptic and mood-stabilizing drug utilized in a clinic for nearly 50 years, has been shown not only to relieve mania symptom of schizophrenia patients but also to improve agitated symptoms of dementia (Czuczwar and Patsalos, 2001; Perucca, 2002; Bauer and Mitchner, 2004). In addition, the study found that VPA is a selective inhibitor of glycogen synthase kinase-3β (GSK-3β; Chen et al., 1999). It has been proposed that GSK-3β may play a pivotal role in AD pathogenesis (Williams et al., 2002; Yang et al., 2017). Accordingly, can VPA protect brain tissue by regulating the activity of GSK-3β? In our previous studies (Qing et al., 2008; Long et al., 2013, 2015), we found that VPA notably inhibited GSK-3β-mediated γ-secretase cleavage of amyloid precursor protein (APP). Such inhibition greatly decreased Aβ generation and SP formation, rescued the neuronal loss and hippocampal synaptic alteration, as well as improved the cognitive performance of AD mice. Our previous studies indicate that VPA is an effective drug for the prevention and treatment of AD.

Interestingly, it has also been demonstrated that VPA can regulate canonical Wnt/β-catenin signaling pathway (Phiel et al., 2003). The Wnt signaling pathway is subdivided into β-catenin dependent (canonical) and β-catenin independent (noncanonical) signaling pathways. It can be activated when a Wnt ligand binding to the seven-pass transmembrane receptor Frizzled, which may invoke canonical or non-canonical signaling cascades (Gordon and Nusse, 2006). The Wnt signaling cascade not only controls the neural development during embryogenesis, but also plays an important role in the adult nervous system, regulating the formation of synaptic contacts, neurotransmission, and plasticity (Inestrosa and Arenas, 2010; Inestrosa and Varela-Nallar, 2015). In addition, the Wnt signaling pathway has been more recently demonstrated to regulate hippocampal neurogenesis in the adult brain (Varela-Nallar and Inestrosa N, 2013).

Our previous experiments suggested that hippocampal neurogenesis declines with age and that AD has profound influences on the regulation of hippocampal neurogenesis in adult brain, which results in reduced production of nerve progenitor cells and inhibition of the neuron differentiation and maturation (Zeng et al., 2016). Given that VPA activates the Wnt signaling pathway (Phiel et al., 2003), we investigated whether or not VPA administration affects hippocampal neurogenesis in a mouse model of AD. In the present study, APP/PS1/nestin-GFP triple transgenic (3xTgAD) mice, a well-characterized mouse model of AD which expresses a green fluorescent protein (GFP) under the control of the nestin promoter, were used for experimental research. Our data showed that VPA promoted cell proliferation and the generation of newborn mature granule cells, increased the density of immature neurons in 3xTgAD mice. Furthermore, VPA promoted the activation of the Wnt signaling pathway in the hippocampus and increased the expression of the procedural Wnt target gene NeuroD1, potentially suggesting that VPA is a promising therapeutic approach for AD prevention and treatment.

## Materials and Methods

### Animals and VPA Treatment

The 3xTgAD mice were obtained as previously described (Zeng et al., 2016). Mice were exposed to 12 h of light/12 h of dark cycle at a constant temperature, and ate and drank freely. All the experimental programs for mice were approved by the Ethics Committee of Animal Laboratory of Chongqing Medical University and carried out in accordance with international standards. Every effort was made to minimize the number of animals used and their suffering. In this study, mice aged 2 months and 6 months were used to represent the early and late stages of Aβ deposition, respectively. The mice in VPA-treated group were intraperitoneally injected with 30 mg/kg body weight VPA, daily, for 4 weeks, while the control group mice were injected with the same amount of saline (*n* = 10 mice/group). After a 4-week treatment, the age of mice was 3 months or 7 months old, so the mice were divided into a 3-month group and a 7-month group.

### Morris Water Maze

The water maze test was carried out in a pool with a diameter of 1.2 m. A 9 cm diameter platform was placed in the southeast quadrant. The experiment includes a visual platform test on the first day and a 5-day hidden platform test in the second to sixth day, as well as a probe trial after the last hidden platform test. In the visible platform test, mice were tested for five contiguous trials with an intertrial interval of 30 min. In the hidden platform tests, mice were trained for six trials with an intertrial interval of 1 h. Mice movement was tracked with a VideoMot2 image analyzer (TSE Systems, China).

### 5-Bromo-2-Deoxyuridine Labeling

Newborn cells were labeled by intraperitoneal injection 5-bromo-2-deoxyuridine (BrdU, Sigma, St. Louis, MO, USA) dissolved in 0.1 M phosphate buffer. BrdU was injected intraperitoneally 33.3 mg/kg every 3 h, three times in total. The brains were taken from mice after 3 h of BrdU injection. BrdU was visualized as described in the immunohistochemistry section.

### Tissue Preparation for Histological Examination

Three hours after BrdU injection, the mice were deeply anesthetized with chloral hydrate and perfused with cold 0.9% saline solution. After that, brain tissues were immobilized in 4% polyformaldehyde (PFA) overnight at 4°C, dehydrated in 0.1 M phosphate buffer saline (PBS, pH 7.2) in 30% sucrose until they sank and embedded in tissue-embedded media (OCT, SAKURA Tissue-Tek, JP). Coronary hippocampus serial sections (10 microns) were obtained by cryosurgery (CM1860, Leica, GER). All sections containing hippocampus were collected sequentially in the 24-well plate containing 0.01 M PBS.

### Immunofluorescence Staining

Free-floating sections were used for immunofluorescence labeling. For general immunofluorescence staining, all sections were washed three times with 0.01 M PBS and blocked for 30 min at 37°C by 0.3% Triton X-100 with 5% fetal bovine serum (HyClone, Logan, Utah). Next, sections were incubated with primary antibodies overnight at 4°C and then tagged with Cy3-conjugated second antibody (1:200, Beyotime, Shanghai, China) at 37°C for 30 min. The nuclei were visualized by DAPI (Beyotime) staining after immunofluorescence labeling. Finally, the sections were mounted on the slides and covered with cover slides for the confocal microscope. For BrdU staining, the DNA denaturation step of 2 N HCl (30 min, 37°C water bath) was added before blocking, so that BrdU could be incorporated into the DNA of mitotic cells.

The main antibodies used in this study were as follows: mouse anti-BrdU (1:200, Abcam) for proliferating cells, rabbit anti-doublecortin (anti-DCX; 1:200, CST, Boston, MA, USA), mouse anti-GFAP (1:200, CST) for astrocyte, and rabbit anti-NeuN (1:200, Abcam, Cambridge, ENG) for mature cells. For secondary antibodies, Cy3 conjugate (1:200, Beyotime) antibodies were used. The images were captured on Nikon A1R + laser scanning confocal microscope (Nikon, Tokyo, JP). Quantification and image analysis referred to the previous description (Zeng et al., 2016).

### Golgi-Cox Staining

Manufacturer’s instructions (FD Rapid GolgiStain™ Kit, FD Neurotechnologies, Inc., USA) were strictly followed. The brains of mice were quickly removed from the skull without perfusion after deeply anesthetized with chloral hydrate, and through it, immersed in an impregnating solution which mixed with the same amount of solution A and B, containing potassium dichromate, potassium chromate and mercury chloride, after that remained in darkness at room temperature for 2 weeks. Next, the brains were transferred to solution C and stored in darkness for at least 72 h. Coronal serial sections (100 μm) were cut with a freezing microtome. The slices were mounted onto gelatinized slides, followed by a description of dyeing steps; After that images were analyzed by a Leica DM 2000 microscope (Leica, Wetzlar, GER). The morphological classifications and the maturity of spines were based on Irwin et al. (2002). The number of spines was counted under a 100× objective lens along the entire dendrite. Dendritic spines were obtained in the distal at least 100 μm of the dendrite. The number of spines was quantified for every 25-μm segment along the entire visible length of the dendrite. Spines less than about 0.4 μm in profile length were not counted; these spines were usually those projecting at oblique angles and partially obscured by overlap with the dendritic shaft. At least five neurons from five different tissue sections were chosen for statistical analysis. The number of spines per μm of dendrite was determined.

### Western Blot

Frozen hippocampal tissue samples were lysed in cold RIPA pyrolysis buffer and PMSF. The pyrolysis products were analyzed by 10% TIS glycine SDS-PAGE electrophoresis. The sample peptide was then electro-transferred to a polyvinylidene fluoride (PVDF) membrane (Millipore, USA) using a water bath transfer device (Bio-Rad, Hercules, CA, USA). Targeting peptides were detected with Anti-GSK-3β (1:1,000 dilution, Abcam, Cambridge, MA, USA), anti-pSer9-GSK-3beta (1:500 dilution, Santa Cruz, CA, USA), rabbit anti-beta-catenin (Cell Signaling Technology, 8480, 1:1,000) and rabbit anti-Wnt3a, rabbit anti-nerve D1 (affinity biology company, Canada, 1:1,000). β-actin was used as internal control. Peroxidase-coupled secondary antibodies (1:10,000, Beyotime, Shanghai, China) were incubated at 37°C for 1 h. The immunoreaction bands were observed by enhanced chemiluminescence (ECL, Thermo Science, IN, USA) by exposure to X-ray film (Fuji, JP) and then quantified by Quantity one software (version 4.6.2, Bio-Rad, Hercules, CA, USA).

### Statistical Analysis

All statistical analyses were carried out by SPSS software (version 18.0). The data are expressed as the mean ± standard error of the mean (SEM). Two-way analysis of variance (ANOVA) was used for statistical comparison, followed by Bonferroni’s post-test with multiple comparisons. All counts are made by the same person who is blindly experimentally conditioned. Quantitative analysis was carried out with the help of IMAGE software (version 1.44 P, USA). The difference was significant (*P* < 0.05).

## Results

### VPA Treatment Significantly Improves Cognitive Impairments in 3xTgAD Mice

**Figure 1:**
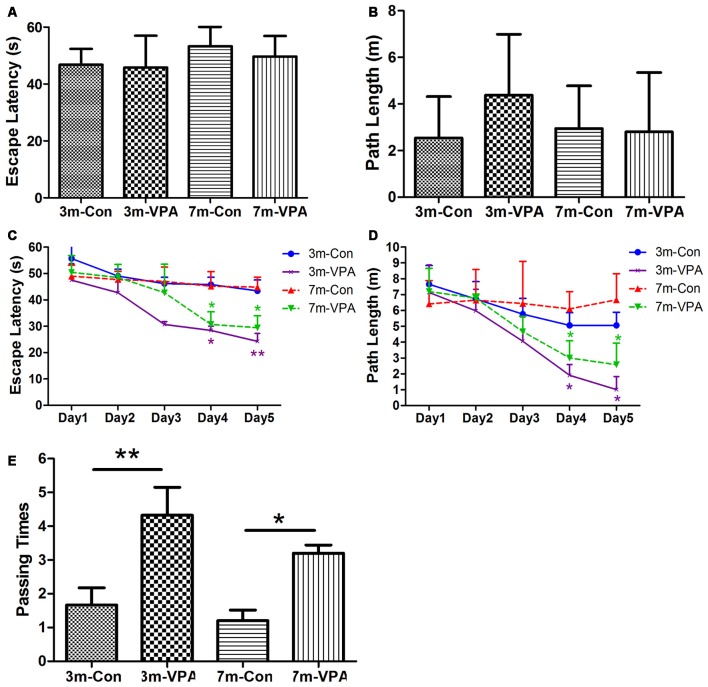
Valproic acid (VPA) improves cognitive ability in 3xTgAD mice. **(A,B)** Visible platform tests. The VPA-treated and control (saline-treated) Alzheimer’s disease (AD) mice exhibited a similar escape latency **(A)** and path length **(B)**. **(C,D)** Hidden platform tests. The VPA-treated AD mice showed a shorter escape latency **(C)** and path length **(D)** on the fourth and fifth days. **(E)** Probe trial. The VPA-treated mice showed significantly more passing times in the target section where the platform was previously located than controls. *P < 0.05, **P < 0.01.

To evaluate whether VPA positively affects cognitive function in a mouse model of AD, we used 3xTgAD mice and treated with VPA for 4 weeks. We evaluated spatial learning and memory of the 3xTgAD mice by using the Morris water maze (MWM) test, a well-established test for spatial learning and memory. We found that in visible platform tests, both in 3-month and 7-month group mice, VPA-treated and saline-treated mice had a similar escape latency (3-month: 45.80 ± 4.56 and 46.76 ± 2.30 s, *P* > 0.05; 7-month: 49.70 ± 2.94 and 53.25 ± 3.15 s, *P* > 0.05; Figure 1A) and path length (3-month: 4.38 ± 1.06 and 2.51 ± 0.72 m, *P* > 0.05; 7-month: 2.81 ± 1.04 and 2.95 ± 0.75 m, *P* > 0.05; Figure 1B), indicating that VPA did not affect the vision or swimming ability of mice.

In the hidden platform tests, VPA-treated mice showed reduced escape latency compared to saline-treated controls on the fourth and fifth days of training, especially in 3 m group mice. The escape latency on the fourth and fifth day of VPA-treated mice was shorter (3-month: 28.42 ± 1.60 and 24.27 ± 2.99 s; 7-month: 30.66 ± 4.82 and 29.42 ± 4.55 s) than that in saline-treated mice (3-month: 45.83 ± 2.72 and 43.42 ± 4.15 s; *P* < 0.01; 7-month: 45.18 ± 5.55 and 44.83 ± 3.80 s; *P* < 0.05; Figure 1C). The path length of VPA-treated mice on the fourth and fifth day was also shorter (3-month: 1.91 ± 0.69 and 1.02 ± 0.81 m; 7-month: 3.01 ± 1.08 and 2.58 ± 1.37 m) than that of saline-treated mice (3-month: 5.07 ± 0.80 and 5.07 ± 0.82 m; *P* < 0.05; 7-month: 6.10 ± 1.09 and 6.67 ± 1.64 m; *P* < 0.05; Figure 1D).

In the probe trial on the last day of testing, the platform was removed. The analysis revealed that VPA-treated mice showed significantly more passing times in the target section where the platform was previously located than saline-treated mice (3-month: 4.33 ± 0.82 and 1.67 ± 0.51; *P* < 0.01; 7-month: 3.20 ± 0.24 and 1.20 ± 0.32; *P* < 0.05; Figure 1E).

### VPA Promotes the Proliferation of Neural Stem Cells in the Hippocampus of 3xTgAD Mice

**Figure 2:**
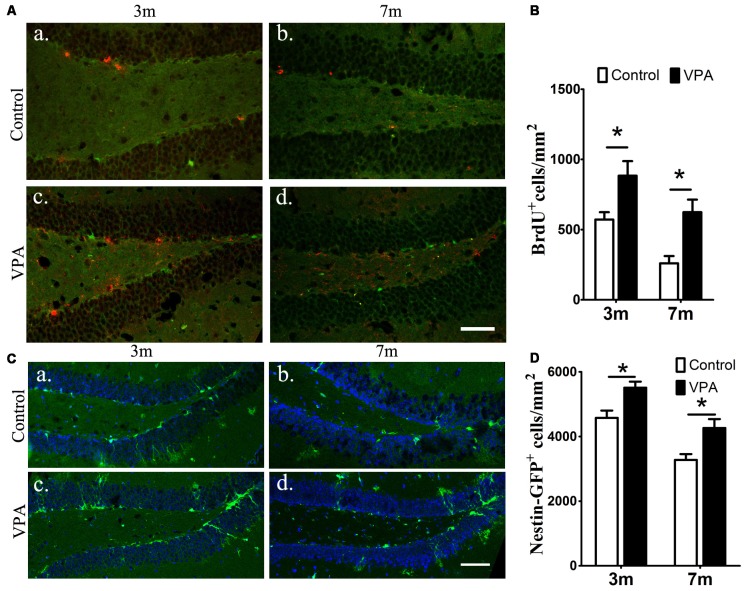
VPA promotes the proliferation of neural stem cells (NSCs). Immunofluorescence staining of 5-bromo-2-deoxyuridine (BrdU^+^) cells (red) in the subgranular zone (SGZ) of the dentate gyrus (DG; **Aa–d**). Quantification of BrdU^+^ cells; VPA-treated mice had more BrdU^+^ cells than controls **(B)**. Expression of nestin-green fluorescent protein (GFP; green) in the SGZ of the DG. Nuclei are stained in blue with DAPI **(Ca–d)**. Quantification of nestin-GFP^+^ cells; VPA-treated mice had more nestin-GFP^+^ cells than controls **(D)**. *P < 0.05, scale bar = 50 μm.

To examine the effect of VPA on progenitor cell proliferation, we used BrdU labeling. BrdU is a marker of proliferating cells, which incorporates into cells that undergo active dividing in the SGZ of the DG of the hippocampus (Taupin, 2007). Notably, a significantly higher number of proliferating cells are present in VPA-treated mice than that in the saline-treated mice (*P* < 0.05, Figures 2Aa–d,B). To confirm if the NSC pool is changed, we quantified the number of nestin-GFP positive cells, which serves as a marker of NSCs (Mignone et al., 2004; Bernal and Arranz, 2018). We found that the number of nestin-GFP positive cells in VPA-treated mice was higher than that in saline-treated control mice at both 3-month and 7-month groups (*P* < 0.05, Figures 2Ca–d,D). These results suggest that VPA treatment increases the number of new neurons and NSCs and promotes NSCs proliferation.

### VPA Facilitates the Differentiation of Neural Stem Cells in the Hippocampus of 3xTgAD Mice

**Figure 3:**
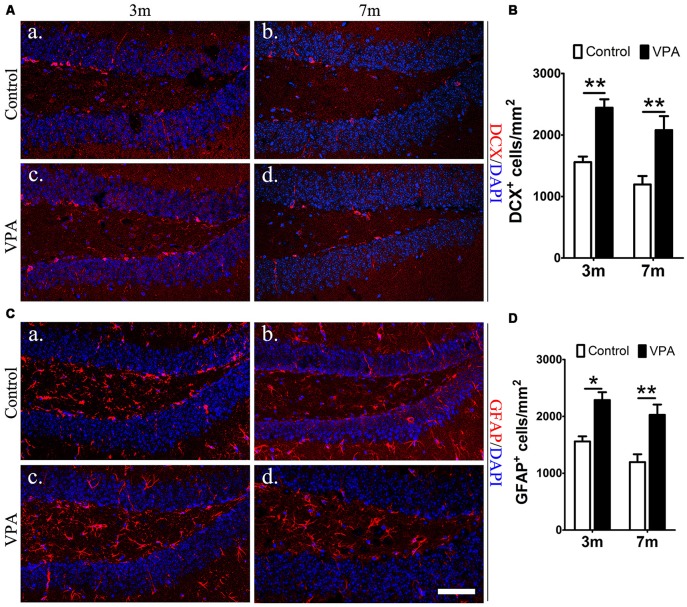
VPA facilitates the differentiation of NSCs. Immunofluorescence staining of doublecortin (DCX^+^) cells (red) in the SGZ of the DG **(Aa–d)**. Quantification of DCX^+^ cells; the number of DCX^+^ cells increased after VPA treatment **(B)**. Immunofluorescence staining of GFAP^+^ cells (red) in the SGZ of the DG **(Ca–d)**. Quantification of GFAP^+^ cells; the number of GFAP^+^ cells increased after VPA treatment. Nuclei are stained in blue with DAPI **(D)**. *P < 0.05, **P < 0.01, scale bar = 50 μm.

To explore whether VPA affects neuronal differentiation, we analyzed the expression of DCX (a marker for immature neurons) and GFAP (a marker for astrocytes and NSCs) in the DG of mice. Compared with saline-treated mice, VPA-treated mice showed a significant increase in the number of DCX^+^ cells at both 3-month and 7-month groups (*P* < 0.01, Figures 3Aa–d,B). Consistent results were obtained from GFAP immunofluorescence staining (*P* < 0.05, Figures 3Ca–d,D). Since GFAP is also a marker of NSCs, it is uncertain whether the GFAP^+^Nestin^−^GFP^+^ cells that we have identified are hypertrophic NPCs or reactive astrocytes. Morphologically, GFAP^+^ cells in VPA-treated mice had larger cell bodies and extended dendrites in the granule cell layer, which indicates that these positive cells were more likely to be reactive astrocytes with stem cell properties, namely, potential DG NSCs. These results show that VPA has the ability to enhance the capacity of NSCs to differentiate into neural precursors and may activate astrocytes, resulting in reactive astrocytes with stem cell qualities.

### VPA Promotes the Maturation of Neurons in the Hippocampus of 3xTgAD Mice

**Figure 4:**
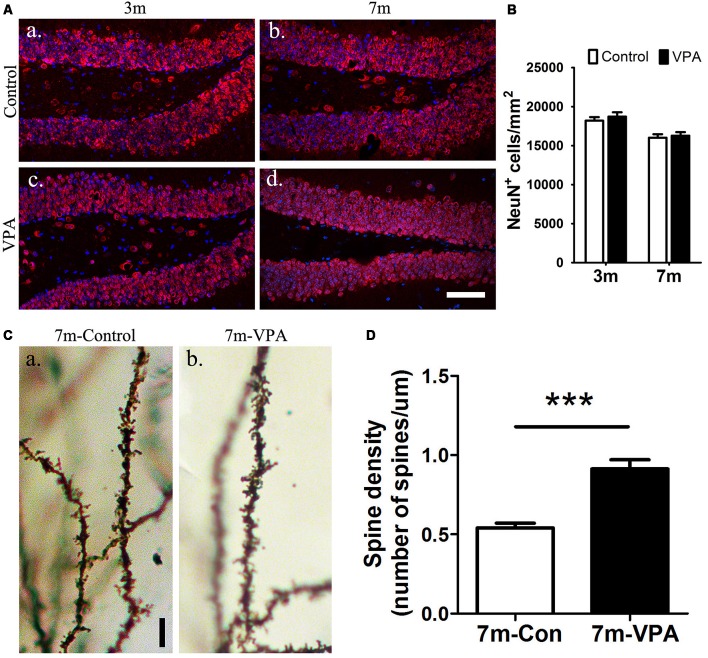
VPA promotes the maturation of neurons. Immunofluorescence staining of NeuN^+^ cells (red) in the SGZ of the DG; scale bar = 50 μm **(Aa–d)**. Statistical analysis showed that there were no obvious differences between the VPA-treated mice and controls, *P* > 0.05 **(B)**. Golgi–Cox-stained dendritic branches of neurons; scale bar = 25 μm **(Ca,b)**. Analysis of spine density; the VPA-treated mice showed an obviously higher spine density than controls **(D)**. ***P < 0.001.

To explore the impact of VPA on the maturation of neurons, the expression of NeuN (a specific marker for mature neurons) were detected. Statistical analysis showed that there were no obvious differences between the VPA-treated mice and saline-treated controls in NeuN expression at both 3-month and 7-month group mice (*P* > 0.05, Figures 4Aa–d,B). However, the VPA-treated mice showed more morphologically intact and homogeneous staining than the saline-treated controls. To further test the consequences of this difference in staining, we used an established Golgi-Cox staining method to analyze the dendritic spines of neuronal cells in the DG, which can reflect the physiological maturity of neurons. Compared with saline-treated mice, VPA-treated mice exhibited an increase in spine density (*P* < 0.001, Figures 4Ca,b,D). These results indicate that VPA treatment has little influence on the survival of neurons but may promote the maturation of neurons to some degree.

### VPA Inhibits the Formation of Senile Plaques

**Figure 5:**
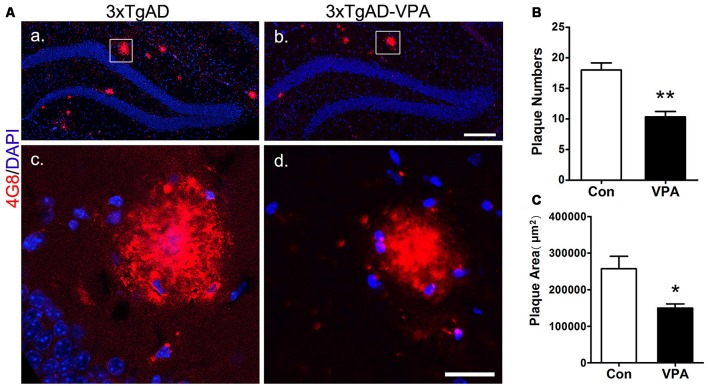
VPA inhibits the formation of amyloid β (Aβ) plaques in AD mice. Immunofluorescence staining of Aβ plaques (4G8, red) in the hippocampus of brains; scale bar = 250 μm **(Aa,b)**. Higher magnifications of the squared areas in **(Aa,b)**, respectively. Scale bar = 25 μm **(Ac,d)**. **(B,C)** Quantitative analyses of the plaque number and plaque area in the hippocampus of VPA-treated mice and controls. The plaque number **(B)** and plaque area **(C)** were significantly reduced by VPA treatment. *P < 0.05, **P < 0.01.

To investigate whether VPA treatment might lead to pathological alterations in the AD brain, 4G8 immunostaining was used to detect amyloid plaque formation in the hippocampus (Figures 5Aa–d). In the 3-month group mice, hardly any amyloid plaques were detected. In the 7-month group mice, quantitative analysis of amyloid plaques showed a significant effect of VPA on plaque number and plaque area. Compared with 4 weeks of saline treatment, 4 weeks of VPA treatment reduced the plaque number and plaque area (Figures 5B,C). These data suggest that VPA inhibits the formation of SPs.

### VPA Inhibits the Activity of GSK-3β and Stimulates the Wnt/β-Catenin Signaling Pathway

**Figure 6:**
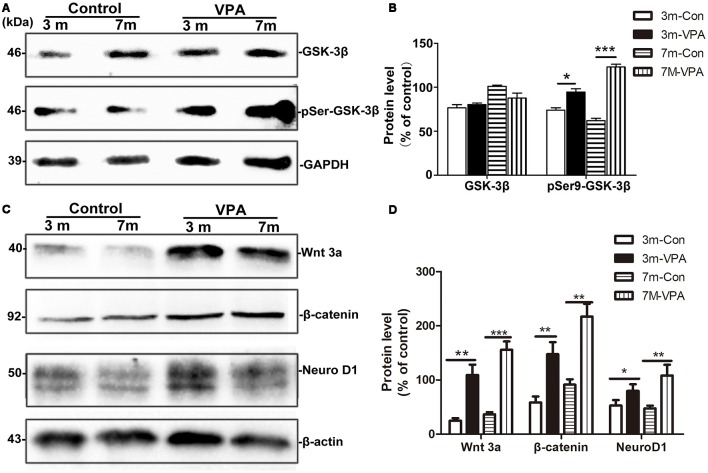
VPA inhibits glycogen synthase kinase-3β (GSK-3β) activity and stimulates the Wnt/β-catenin signaling pathway. **(A)** Western blot analysis of total GSK-3β and p-GSK-3β^S9^ (pSer9). GAPDH was used as an internal control. **(B)** Densitometric analysis of total GSK-3β and p-GSK-3β^S9^ (pSer9). VPA increased pSer9-GSK-3β levels but not total GSK-3β levels in AD mice. **(C)** Immunoblots of β-catenin, Wnt3a and NeuroD1; β-actin was used as an internal control. **(D)** Densitometric analysis of β-catenin, Wnt3a and NeuroD1 normalized to β-actin. VPA increased β-catenin, Wnt3a and NeuroD1 levels in AD mice. *P < 0.05, **P < 0.001, ***P < 0.0001.

GSK-3β is known to regulate Aβ formation (Llorens-Martín et al., 2014). The activity of GSK-3β is most commonly inhibited when Ser9 residues were phosphorylated (Woodgett, 1994). We evaluated the effect of VPA on GSK-3β and pSer9-GSK-3β in the hippocampus of mice. No differences were found in total GSK-3β protein levels between the VPA-treated and saline-treated groups, while significantly higher pSer9-GSK-3β protein levels were detected in the VPA-treated mice than in the saline-treated mice (Figures 6A,B).

GSK-3β is an important factor of the Wnt/β-catenin signaling pathway (Frame and Cohen, 2001). Compared with the control mice, the β-catenin level in the hippocampus of VPA-treated animals increased significantly, with a concomitant increase in the level of the inactive form of Wnt3a. In addition, significantly higher NeuroD1 levels were observed in the hippocampus of VPA-treated mice than in that of control mice (Figures 6C,D). Altogether, these findings indicate that VPA stimulates the activation of the Wnt/β-catenin signaling pathway in the hippocampus of 3xTgAD mice.

## Discussion

AD is the most common chronic progressive neurodegenerative disease in the elderly, with progressive memory decline, cognitive impairment and abnormal personality and behavior as the main clinical manifestation. The early symptoms and learning and memory impairments in AD suggest that the hippocampus is damaged. Numerous studies have indicated that changes in hippocampal neurogenesis in AD contribute to the plasticity of the hippocampus and to some hippocampal processes, including spatial learning and memory (Becker et al., 2007; Deng et al., 2010; Lazarov et al., 2010). Therefore, the search for drugs or approaches to stimulate hippocampal neurogenesis in the AD brain is a tempting research focus, providing a new treatment strategy for AD.

In recent years, studies have found that VPA, an anti-epileptic drug applied in the clinic, can relieve the pathological features of AD to a certain degree (Williams et al., 2002; Tariot et al., 2011; Wang et al., 2014). Here, we evaluated the effect of VPA to activate neurogenesis and demonstrated that VPA can induce the proliferation and generation of new neurons in the adult hippocampus in AD. For this purpose, APP/PS1/nestin-GFP triple transgenic mice, which are not only a well-characterized AD mouse model but also a good mouse model for neurogenesis research due to their expression of green fluorescent protein under the control of the nestin promoter were used in this study. 2 months and 6-months-old mice, representing the early and advanced stages of AD, were chosen to be treated with VPA for 4 weeks. Consistent with previous studies, our results showed that VPA treatment evidently improved the spatial learning and memory abilities of AD model mice (Qing and He et al., 2008). Studies showed that the decline in neurogenesis may lead to some degree of cognitive impairments (Lazarov et al., 2010), and the activation of hippocampal neurogenesis is beneficial to alleviate AD symptoms (Becker et al., 2007).

In the adult DG, new granule cells generate neural progenitor cells that are located between the GCL and the hilus continuously. After activation, these neural progenitor cells give rise to transit-amplifying progenitors or intermediate progenitor cells, which then generate neuroblasts that develop into immature neurons that extend dendrites to the GCL and molecular layer and project their axons to the CA3 region (Zhao et al., 2008). Over the course of several weeks, these newborn neurons mature into functional dentate granule neurons which form synaptic connections and integrate into the hippocampal circuitry (Laplagne et al., 2006).

We determined that 4 weeks of VPA treatment increased the number of new neurons and NSCs and activated the proliferation of progenitor cells in the SGZ. VPA treatment increased the number of cells positive for BrdU, which represents a subtype of progenitors for new neurons (Kempermann et al., 2003). Nestin-GFP^+^ cells, which are progenitors with the ability to proliferate (Namiki et al., 2012), were also found to be obviously increased in VPA-treated mice. These results illustrate that VPA promoted the proliferation of NSCs. Next, VPA treatment was found to increase the number of DCX^+^ cells, which represent postmitotic progenitors and early immature neurons (Brown et al., 2003). GFAP^+^ cells, which are cells with astrocytic properties that can act as a source of new neurons during adult neurogenesis (Doetsch et al., 1997), were also found to be in higher numbers in VPA-treated mice. These data suggest that VPA promoted the differentiation of NSCs. Accordingly, to explore the impact of VPA on the maturation of neurons, we detected the expression of NeuN (a specific marker for mature neurons) and analyzed the dendritic spine density of neuronal cells in the DG, which can reflect the physiological maturity of neurons. These results indicate that VPA treatment has little influence on the survival of neurons but may promote, to some degree, the maturation of neurons. To an extent, newborn neurons compensate for the loss of neurons in AD, rebuilding the neural pathways and thus improving the state of AD (Haughey et al., 2002), which stimulates hippocampal neurogenesis in AD mice, embodied by the promotion of cell proliferation, differentiation and maturation. A study found that after Aβ deposition, fewer NSCs and new neurons were found in the hippocampus, and hippocampal neurogenesis was reduced (Taniuchi et al., 2007). Moreover, consistent with previous studies that showed that VPA inhibited Aβ production in AD mice (Su et al., 2004), we found that 4 weeks of VPA treatment remarkably reduced the number and area of plaques in the AD brain.

GSK-3β is closely related to the pathological alterations of AD. The abnormal activation or overexpression of GSK-3β may result in increased formation of Aβ, excessive phosphorylation of tau and large loss of neurons. GSK-3β plays an important role in regulating cell proliferation and differentiation (Barghorn et al., 2000; Friedland et al., 2001; Sirerol-Piquer et al., 2011). *In vitro* and *in vivo* studies have been reported that GSK-3β inhibition stimulates neurogenesis. Lithium, a GSK-3β inhibitor, can induce the proliferation of adult hippocampal progenitor cells (Wexler et al., 2008), and treatment with lithium-induced proliferation and neuronal fate specification in the hippocampus of the AD mouse model (Fiorentini et al., 2010). In addition, impaired neurogenesis was observed in a GSK-3β knock-in mouse carrying mutations to block inhibitory phosphorylation of the kinase (Eom and Jope, 2009) and in mice overexpressing GSK-3β (Kimura et al., 2011), which also show morphological alterations in newborn neurons (Llorens-Martín et al., 2013). VPA was confirmed to be a selective GSK-3β inhibitor (Williams et al., 2002). In this study, we found that VPA inhibited the activity of GSK-3β by regulating its phosphorylation state. However, compared to saline-treated mice, VPA-treated mice showed significantly higher protein levels of pSer9-GSK-3β. Our findings demonstrate that VPA promoted hippocampal neurogenesis *via* inhibition of GSK-3β.

GSK-3β is an important component of Wnt signaling pathway. The key regulatory factor of Wnt/β-catenin neurogenesis can induce the differentiation of adult hippocampal NSCs (Lie et al., 2005). The Wnt/β-catenin pathway participates in the regulation of NPC proliferation and differentiation (Yu et al., 2006). It has been reported that the Wnt signaling cascade has been identified as a regulator of self-renewal and proliferation among a variety of stem and progenitor cell populations. Wnt plays multiple roles in early development, including the differentiation of precursor cells. Wnt signaling cascades participate in the formation of neuronal circuits, playing roles in dendrite and axon development, dendritic spine formation and synaptogenesis. In the adult brain, Wnts control hippocampal plasticity, regulating synaptic transmission and neurogenesis (Inestrosa and Arenas, 2010; Inestrosa and Varela-Nallar, 2015; Mohammed et al., 2016). Increasing evidence suggests a role for unbalanced Wnt/β-catenin signaling in AD and impaired neuronal plasticity. Supporting evidence demonstrating the critical role that Wnt/β-catenin signaling can increase the proliferation and self-renewal of NSCs, and the down-regulation of Wnt signal can inhibit hippocampal neurogenesis (Pei et al., 2012; Chen et al., 2018). The majority of the empirical evidence regarding Wnt/β-catenin signaling in AD points to attenuation of this pathway, leading to failure to downregulate GSK-3 activity and subsequent hyperphosphorylation of tau (Boonen et al., 2009). Furthermore, Wnt signaling loss can accelerate the appearance of the neuropathological hallmarks of AD (Tapia-Rojas and Inestrosa, 2018). Here, we determined that VPA treatment increased the expression of β-catenin protein, Wnt3a, and NeuroD1 which is Wnt target gene and needed for the survival and maturation of adult-born neurons (Gao et al., 2009; Kuwabara et al., 2009). Consequently, the effect of VPA might be mediated by activating Wnt signaling pathway and increased expression of NeuroD1 in AD model mice with decreased proliferation of SGZ and decreased differentiation of neural progenitor cells into neurons (Abbott et al., 2013; Varela-Nallar et al., 2014).

In conclusion, this study demonstrated that VPA has a far-reaching effect on the regulation of hippocampal neurogenesis in adult AD mice, leading to an increase in the generation of neural progenitor cells and promoting the differentiation and maturation of neurons. Meanwhile, VPA has the ability to promote the cognitive function of AD mice and reduce Aβ production. The possible mechanism that may explain all of the above results is that VPA inhibits GSK-3β and activates the Wnt/β-catenin signaling pathway, thereby enhancing hippocampal neurogenesis and ameliorating the cognitive impairments of AD mice.

## Data Availability

The datasets generated for this study are available on request to the corresponding author.

## Author Contributions

All authors have contributed to the work and agree with the presented findings.

## Conflict of Interest Statement

The authors declare that the research was conducted in the absence of any commercial or financial relationships that could be construed as a potential conflict of interest.

## Abbreviations

3xTgAD: APP/PS1/nestin-GFP triple transgenic mice
AD: Alzheimer’s disease
ANOVA: analysis of variance
APP: amyloid precursor protein
Aβ: amyloid-β
BrdU: 5-bromo-2-deoxyuridine
DCX: doublecortin
DG: dentate gyrus
GSK-3: glycogen synthase kinase
LVs: lateral ventricles
MWM: Morris water maze
NFTs: neurofibrillary tangles
NSCs: neural stem cells
PBS: phosphate-buffered saline
PS1: presenilin-1
PVDF: polyvinylidene fluoride
SEM: standard error of the mean
SGZ: subgranular zone
SVZ: subventricular zone
VPA: valproic acid.
